# Neurovascular pericytes are susceptible to infection by JC polyomavirus

**DOI:** 10.1128/jvi.00616-25

**Published:** 2025-08-14

**Authors:** Bethany A. O'Hara, Kaitlin Garabian, Wenqing Yuan, Walter J. Atwood, Sheila A. Haley

**Affiliations:** 1 Department of Cell Biology, Biochemistry, and Molecular Biology, Brown University, Providence, Rhode Island, USA; International Centre for Genetic Engineering and Biotechnology, Trieste, Italy

**Keywords:** host range, pericytes, blood brain barrier, progressive multifocal leukoencephalopathy, JCPyV, polyomavirus

## Abstract

Progressive multifocal leukoencephalopathy (PML), an often-fatal neurodegenerative disease, is caused by the neuroinvasive polyomavirus JCPyV. Peripheral organs, including the kidney, are the site of lifelong persistent infections that are asymptomatic. In a subset of immunosuppressed or immunomodulated patients, the virus invades the central nervous system infecting oligodendrocytes, which leads to the multifocal white matter disease known as PML. The mechanisms that lead to neuroinvasion by JCPyV have not been well described. The brain is protected from viruses and other pathogens by physiological barriers, including the blood-cerebrospinal fluid barrier and the blood-brain barrier. To better understand the mechanism by which the virus breaches these barriers, we focused our attention on studying virus interactions with pericytes, which are an essential component of the blood-brain barrier. We found that the virus binds to pericytes in a receptor-dependent manner and that pericytes are susceptible to JCPyV infection. Previous work from our group demonstrated that JCPyV was capable of penetrating an intact endothelial cell barrier. Once across the endothelium, JCPyV would come in direct contact with pericytes, and we hypothesize pericyte infection would amplify and facilitate robust penetration into the brain parenchyma. This is the first demonstration that pericytes, a principal component of the blood-brain barrier, are susceptible to the neuroinvasive human polyomavirus JCPyV.

**IMPORTANCE:**

JCPyV infects at least half the adult population worldwide. An asymptomatic, persistent infection is typically established in the kidney and possibly other peripheral organs. In immunosuppressed individuals, the virus can reactivate and cause progressive multifocal leukoencephalopathy, a deadly disease of the central nervous system (CNS). The pathogenic route the virus takes from the periphery to the CNS is unknown. Here, we demonstrate for the first time that JCPyV can infect human cerebrovascular pericytes, a cell type that contributes to the blood-brain barrier. This observation suggests that the virus could use the pericytes as a means to penetrate the blood-brain barrier to reach its pathogenic targets in the brain parenchyma.

## INTRODUCTION

JCPyV is a ubiquitous, human-specific polyomavirus that infects up to 80% of the human population worldwide (1–4). It is a small, nonenveloped virus with a double-stranded DNA genome of 5,130 bp. The bidirectional genome is divided into an early region that encodes the nonstructural small and large T antigens and the late region that encodes three capsid proteins, VP1, VP2, and VP3, as well as agnoprotein and two microRNAs (5–7). These two regions are divided by the non-coding control region, which supplies the origin of replication and transcription factor binding sites. Interaction of the virus with host cells is mediated by the binding of VP1 to the α2,6-linked sialic acid-containing lactoseries tetrasaccharide c (LSTc) motif on the cell surface (8, 9). Subsequent viral entry is by clathrin-mediated endocytosis, via interaction with the 5-hydroxytryptamine 2 (5-HT_2_) family of serotonin receptors (10–14). Additionally, infectious JCPyV can be packaged and secreted in extracellular vesicles (EVs) (15).

Initial infection is thought to be established through the fecal-oral route and is typically followed by the establishment of an asymptomatic, persistent infection in the peripheral organs, including the kidney and bladder (16, 17). However, in patients who are severely immunocompromised or who are treated with certain immunomodulatory drugs, JCPyV reactivates and migrates from its sites of latency in the peripheral tissues, enters the bloodstream (secondary viremia), and can cause progressive multifocal leukoencephalopathy (PML), a devastating disease of the central nervous system (CNS) (18, 19). Once in the brain, the virus infects oligodendrocytes, astrocytes, and, in some cases, neurons (20). The infection and subsequent lytic destruction of the myelin-producing oligodendrocytes triggers demyelination and the formation of white matter lesions, the hallmarks of PML (21–23). PML is generally not diagnosed until late in disease and, as a result, has a high degree of morbidity and mortality. There are no approved therapeutics to treat PML, and restoration of immune function remains the only clinical option (24–26). Immune reconstitution can also lead to immune reconstitution inflammatory syndrome, an inflammatory syndrome that contributes to disease progression if it is not managed appropriately (27–29). A significant obstacle to developing treatments for PML is that JCPyV only infects human cells, and therefore, there are no robust *in vivo* animal studies. However, progress has been made in the development of mouse models of PML, including using mouse polyomavirus as a surrogate to study mechanisms of disease and reactivation (30–33).

A fundamental question in the study of JCPyV pathogenesis is how the virus surmounts the physiological barriers that protect the CNS from the periphery. In order to infect glial cells, the main target of pathogenesis, JCPyV must overcome multiple structures that exclude pathogens from the brain, including the blood-brain barrier (BBB). The BBB is made up of a monolayer of neuroendothelium surrounded by pericytes and astrocytic endfeet (see the model in Fig. 6) (34). Together, these cells compose the neurovascular unit (NVU). It has long been observed that PML lesions frequently occur along the vasculature (21, 22, 35), suggesting that JCPyV may enter the brain through the BBB to cause disease. In our recent experiments, we have observed that brain microvascular endothelial cells are not permissive for productive JCPyV infection, although a small amount of virus or virus associated with extracellular vesicles can penetrate the endothelial barrier (36). Therefore, we examined whether the pericytes of the NVU could be a potential target of infection and a pathway for JCPyV to invade the CNS.

Brain vascular pericytes are specialized mural cells that help maintain the integrity of the blood-brain barrier. The pericytes are embedded in the basement membrane that is shared with the neuroendothelium and cover the majority of the abluminal side of the brain microvasculature (37). These cells, in turn, are covered by astrocytic endfeet, a primary target of JCPyV infection and pathogenesis. Roles of pericytes include maintenance of endothelial cell-cell junctions, secretion of growth factors and chemokines, clearance of debris, the regulation of BBB permeability and capillary blood flow, and suppression of endocytic activity of the endothelium (38–41). Pericytes also regulate neuroinflammation in the brain following infection and the entry of immune cells into the CNS (42), and pericyte-endothelial cell communication pathways maintain mutual barrier stability and promote cell survival.

Here, we demonstrate that primary human pericytes are susceptible to infection by JCPyV. Pericytes express the binding and internalization receptors known to mediate viral infection, and these cells can support replication and productive infection with both Mad-1 and Mad-4 strains of virus. Infected pericytes also secrete mature infectious virions as well as infectious extracellular vesicles. Our results suggest that the pericytes of the NVU may act as a conduit for JCPyV to invade across the blood-brain barrier and into the brain parenchyma to cause PML.

## MATERIALS AND METHODS

### Cell culture

SVG-A cells, a transformed human glial cell line, were used for reinfection assays and virus propagation. SVG-A cells were cultured in Eagle’s Minimum Essential Media (Corning, #10-010-CM), supplemented with 10% heat-inactivated fetal bovine serum (Atlanta Biologics, #S11150H) and 1% antibiotic-antimycotic (Invitrogen, #15240096). Primary human pericytes were purchased from ScienCell at passage 1 and grown according to the supplier’s protocol using cell line-specific reagents. In brief, culture vessels for pericytes were coated with a 2 µg/mL poly-L-lysine solution (ScienCell, #0-413) prior to plating. Pericytes were maintained in pericyte basal media (ScienCell, #1201), supplemented with 2% heat-inactivated fetal bovine serum, 1% penicillin-streptomycin, and pericyte growth factor (proprietary formulation; ScienCell, #1201). Primary pericytes for experiments were used between passages 3 and 10 and included cells from two different donors.

### Antibodies

To quantify virus following infection, primary antibodies against SV40 VP1 (Pab597) and JCPyV T antigen (Pab962) were used. Pab597 is cross-reactive with JCPyV VP1. Pab597 hybridoma supernatant was used unpurified at a 1:50 dilution overnight at 4°C. Pab962 was a kind gift from the Tevethia laboratory, and hybridoma supernatant was used unpurified at a 1:20 dilution, overnight at 4°C. Alexa Fluor 488 (Invitrogen, #A11001) goat anti-mouse secondary antibodies were used to detect Pab597 and Pab962.

### Virus propagation, purification, and labeling

JCPyV was propagated in SVG-A cells using previously described methodology. In brief, SVG-A cells were cultured and infected with Mad1/SVEΔ (Turbo) at a multiplicity of infection (MOI) of 10. Infection was allowed to spread through the culture for 3 weeks, with weekly media changes. When significant lysis and vacuolization was apparent, the infected culture was collected in whole by scraping. Cells were treated with deoxycholate, sonication, neuraminidase, and repeated freeze-thaw cycles to release virions from the cells. Virions were purified by ultracentrifugation through a 20% sucrose cushion, followed by cesium chloride density gradient ultracentrifugation. Purified virions were collected from the gradient using a syringe, and viral titer was quantified by qPCR comparison to a standard curve. Purified Mad1/SVEΔ was labeled according to the manufacturer’s instructions with Alexa Fluor 488 Succinimidyl Ester Dye (Invitrogen, #A20000).

### Extracellular vesicles

To generate infectious EVs, EVs were concentrated by differential centrifugation of supernatant from infected SVG-A cells. SVG-A cells were infected with purified Mad1/SVEΔ and maintained in vesicle-depleted media for 7 days. At 7 days post-infection, supernatant containing infectious EVs was spun at 300 × *g* in a Sorvall Legend X1R centrifuge (Thermo Fisher Scientific) for 10 min, followed by a 2,000 × *g* spin for 10 min, and two 30 min spins at 10,000 × *g* in a Sorvall Lynx 6000 centrifuge. Supernatant was then transferred to Ultra-Clear tubes (Beckman Coulter) and spun at 100,000 × *g* for 2 h, 9 min in an SW41 Ti rotor (*k*-factor = 124). The pellet was washed with 1× phosphate buffered saline (PBS) and centrifuged for an additional 2 h at 100,000 × *g*. The pellet was suspended in sterile PBSsupplemented with human albumin and trehelose (PBS-HAT) at 1/100th of the original volume. All centrifugation steps were carried out at 4°C, and EVs were stored under liquid nitrogen. Supernatants were transferred to a clean tube after each centrifugation step. EV preparations were characterized by nanoparticle tracking analysis using a ZetaView Quatt (Particle Metrix). To confirm that EVs were infectious, EVs were used to infect SVG-A cells and scored for VP1^+^ nuclei. The concentration of protected genome copies per milliliter of virus in EV preparations was determined by quantitative PCR using a VP2 primer-probe set and comparison to a standard curve. Specific conditions are detailed in the quantitative PCR section.

### Flow cytometry

Primary pericyte cultures were cultured according to the supplier’s recommended protocols, using cell line-appropriate reagents. For flow cytometry, culture media were removed and cells were washed twice with 1× PBS, followed by incubation at 37°C for 10 min in Cellstripper (Corning, #25-056-CI). Cell suspensions were centrifuged for 5 min at 1,000 rpm to pellet, and resuspended in ice-cold 1× PBS at 1 × 10⁶ cells/mL. All virus binding and internalization experiments were carried out using Alexa Fluor 488-labeled JCPyV. Data are reported with the internalized signal subtracted (binding), or post-trypan quench (internalization). The use of trypan blue as a quencher for AF488 allows us to clearly distinguish between internalized and externally bound JCPyV.

#### Virus binding

A total of 100 µL of 1 × 10⁶ cells/mL cell suspension was used per sample, in triplicate. Samples were incubated in the dark on ice for 1 h with Mad1/SVEΔ-AF488 (1:100 dilution). After binding, the volume of all samples was raised to 1 mL using ice-cold PBS to wash and remove unbound virus. Cell suspensions were washed and centrifuged for 5 min at 1,000 rpm to pellet, and were resuspended in ice-cold 2% paraformaldehyde (Thermo, #28908). Samples were run using a BD FACSCanto II instrument and analyzed in FlowJo.

#### Competition assay

LSTc (V-labs, Inc.) was reconstituted to 5 mM in 200 µL sterile 1× PBS. A 1:100 dilution of Mad1/SVEΔ-AF488 was incubated on ice with 5 mM LSTc for 1 h in the dark. During incubation, 100 µL of 1 × 10⁶ cells/mL pericyte cell suspension was prechilled on ice. After binding virus and LSTc together for 1 h, chilled cells were pelleted and resuspended in virus-LSTc complex. The complex with cells was incubated for an additional 1 h on ice in the dark. Cell suspensions and complexes were washed and centrifuged for 5 min at 1,000 rpm to pellet, and resuspended in ice-cold 2% paraformaldehyde. Samples were run using a BD FACSCanto II instrument and analyzed in FlowJo.

#### Neuraminidase treatment

Type II neuraminidase (Millipore-Sigma, #N6514-1UN) was used to remove sialic acid from cell membranes. In brief, 100 µL of 1 × 10⁶ cells/mL cell suspension was used per sample, in triplicate. Samples were centrifuged for 5 min at 1,000 rpm to pellet and resuspended in 1× PBS, pH 5.9, containing 1U of neuraminidase. Samples were incubated for 1 h at 37°C. After neuraminidase treatment, samples were washed with 1× PBS and centrifuged for 5 min at 1,000 rpm to pellet, and resuspended in 1× PBS, pH 7.2, followed by either binding with Mad1/SVEΔ-AF488 (1:100 dilution) on ice for 1 h, or incubation with Mad1/SVEΔ-AF488 (1:100 dilution) at 37°C for 1 h to allow virus to internalize. At the end of incubation with virus, cell suspensions were washed and centrifuged for 5 min at 1,000 rpm to pellet, and resuspended in ice-cold 2% paraformaldehyde. Samples were run using a BD FACSCanto II instrument and analyzed in FlowJo.

### Transfections

At 24 h prior to transfection, primary human pericytes and SVG-A cells were plated into six-well, tissue culture-treated dishes (CellTreat, #229105) at a density of 200,000 cells/well and cultured in complete media as appropriate per cell type. Viral DNA from Mad-1, Mad-4, Mad-1/SVEΔ, carried in the BamHI restriction site of pBR322, was used for transfections. DNA was quantified by Qubit using the double stranded DNA (dsDNA) High Sensitivity Quantification Assay (Invitrogen, #Q32851) and digested with BamHI (Promega, #R6021) prior to transfection. Cells were transfected with 2 µg per well using polyethylenimine (PEI, #R6021) at a 3:1 reagent:µg DNA ratio, in antibiotic-free media. Mock transfection controls contained a linearized pBR322 vector without viral DNA. One day following transfection, media were replaced on all samples to remove PEI, and cells were cultured an additional 3 days in complete media. At 4 days post-transfection, cells and supernatants were collected for quantitative PCR to measure virus released into the media, as well as viral protein expression in the cells.

### Infections

Primary pericyte cultures were cultured according to ScienCell recommended protocols, using cell line-appropriate reagents. SVG-A cells were cultured as described above. At 24 h prior to infection, 96-well plates (Corning, #353072) were coated with poly-L-lysine (ScienCell, #0-413). Pericytes and SVG-A cells were seeded at a density of 10,000 cells/cm^2^.

#### Initial infections

Pericytes were infected with either purified Mad1/SVEΔ (Turbo) at an MOI of 10 particles or with infectious extracellular vesicles diluted 1:5 in 1× PBS for 2 h at 37°C. Following incubation with virus or infectious EV, infection media were removed and replaced with complete media as appropriate per cell type. Cells were maintained in complete media for 96 h.

#### Reinfections using supernatant

Supernatant from pericytes at 4 days post-infection was used to reinfect naïve SVG-A cells. Cells were plated at 10,000 cells/cm^2^ in 96-well plates 1 day prior to infection. Supernatant from JCPyV- or EV-infected and uninfected control pericytes was added to SVG-A cells for 2 h at 37°C. Following incubation, pericyte supernatant was removed and replaced with complete media. Cells were maintained in complete media for 96 h.

### Staining and image analysis

Forty-eight or 72 h after initial infection or reinfection using supernatant, pericytes and SVG-A cells were fixed using ice-cold methanol (VP1 stains) or ice-cold ethanol (T antigen stain) for 20 min at −20°C. Cells were rehydrated using room temperature 1× PBS for 20 min, then permeabilized with 1% Triton-X for 10 min at room temperature. Triton-X was removed and primary antibody against VP1 (Pab597) or T antigen (Pab962) was added in 1× PBS with 1% goat serum and 0.1% Triton-X and incubated overnight with rocking at 4°C. Unbound primary antibody was removed with three washes of 1× PBS, followed by co-incubation with Alexa Fluor goat anti-mouse 488 secondary antibody (Invitrogen, #A11001) and 4’,6-diamidino-2-phenylindole (DAPI) (Invitrogen, #62248), both diluted 1:1,000 for 1 h at 37°C. Total cell count (DAPI), as well as T-ag- and VP1-positive nuclei, were quantified using Elements High Content software on a Nikon Ti2-E fluorescence microscope. Results are shown as a percentage of T-ag^+^ or VP1^+^ nuclei compared to total cell count.

For characterization of cultured primary pericytes, cells at passage 3 were grown on coverslips and fixed in 4% paraformaldehyde for 15 min at room temperature. The coverslips were then washed three times for 5 min in 1× PBS and blocked for 1 h at room temperature in blocking buffer (5% normal goat serum and 0.3% Triton X-100 in 1× PBS). The coverslips were stained with primary antibodies: rabbit anti-platelet-derived growth factor receptor-β ((PDGFRβ) (Cell Signaling Technologies, #3169; 1:100), rabbit anti-NG2 (Chemicon, #AB5320; 1:250), and rabbit anti-glial fibrillary acidic protein (GFAP) (Abcam, #ab7260; 1:500), and incubated overnight at 4°C. After overnight incubation, coverslips were washed and then visualized after a 1 h room temperature incubation with a secondary antibody conjugated to Alexa Fluor 594 (Invitrogen, #A11037; 1:2,500). All samples were counterstained with 1 µg/mL DAPI (Thermo, #62248).

### Western blots

Samples of primary human pericytes and astrocytes were lysed on ice for 30 min in RIPA (radioimmunoprecipitation assay buffer; Thermo, #89900) supplemented with complete EDTA-free protease inhibitor cocktail (Sigma/Roche, 11836170001) and 0.1 mM phenylmethylsulfonyl fluoride (PMSF), sonicated, and centrifuged at 10,000 RPM at 4°C for 20 min to pellet debris. Protein content was determined using the Pierce Rapid Gold BCA Protein Assay Kit (Thermo, #A53226). Samples were prepared in 4× Laemmli buffer (Bio-Rad Laboratories, Hercules, CA) and 15% beta mercaptoethanol (BME), boiled at 95°C for 10 min, and loaded in 4%–15% gradient Mini-Protean TGX Stain-Free precast gels (Bio-Rad). Gels were run at 150V and transferred to polyvinylidene difluoride (PVDF) membranes by the semidry transfer method. Blots were blocked in 5% nonfat dried milk/Tris-buffered saline with 0.01% Tween 20 (TBST) for 1 h at room temperature. Primary antibodies (PDGFRβ, CST, #3169, 1:1,000; GFAP, PTG, #16825, 1:5,000; actin loading control, CST, #3700, 1:1,000) were diluted in blocking buffer and incubated overnight at 4°C. Blots were washed three times with TBST and incubated with horseradish peroxidase-conjugated secondary antibodies diluted to 1:5,000 for 1 h at room temperature. The blots were then washed three times with TBST and then incubated with Clarity Max Western ECL Substrate (Bio-Rad, #1705062) according to the manufacturer’s protocol for 5 min and subsequently imaged on a ChemiDoc MP imaging system (Bio-Rad).

### qPCR of JCPyV stocks, infectious EVs, and culture supernatants

To determine copies per microliter in purified virus and infectious EV preparations, a 5 µL sample of virus or infectious EV was treated with DNase 1 (New England Biolabs, #M0303L) to remove free DNA, leaving behind intact extracellular vesicles or virions with intact capsids. DNase-treated samples were extracted using a DNeasy Blood & Tissue Kit (Qiagen, #69581). The extraction step contains a proteinase K digest, which breaks open vesicle membranes as well as the viral capsid, exposing the internal (protected) genome. DNA from extracted samples was analyzed by qPCR, alongside a 10-fold standard curve of Mad1-pBR322 DNA using a primer-probe set targeting JCPyV VP2. Samples were run on a Bio-Rad CFX Maestro and analyzed using CFX Maestro software. Sequences for the VP2 assay primer-probe set used are as follows:

VP2 assay primer-probe set (Custom synthesis, IDT) probe: 5´-/5HEX/TGTTCTCCA/ZEN/CAATCTCCCAGGCTT/3IABkFQ/-3´ primer 1: 5´-CCTGGAGTGAATGCCTTTGT-3´ primer 2: 5´-AGAGGTTAAGGCTGGCAAATC-3´

For genome quantification from transfected cell supernatants, supernatant was collected at 4 days post-transfection and treated with DNase 1 (New England Biolabs) to remove non-encapsidated genomes. The same extraction methods and VP2 primer-probe were used as above for determining viral stock titer. Genome concentration was calculated by comparison to a 10-fold standard curve of Mad1-pBR322 plasmid DNA.

### RT-qPCR of transfected cells and receptor analysis

SVG-A cells and pericytes transfected with JCPyV or empty vector were collected at 4 days post-transfection. Cells were first washed twice in sterile 1× PBS, then dissociated using trypsin (Corning, #25-052-CI). Cells were spun for 5 min at 1,000 rpm and washed an additional time in 1× PBS to remove residual media and trypsin. RNA was extracted using an RNeasy Mini Kit (Qiagen, #74104) and quantified for total RNA per sample by Qubit RNA High Sensitivity Assay Kit (Invitrogen, #Q32852). Equivalent amounts of RNA per sample were used for cDNA synthesis (Bio-Rad, #1708890). cDNA was used to run RT-qPCR on a Bio-Rad CFX Maestro and analyzed using CFX Maestro software. Sequences for virus and glyceraldehyde-3-phosphate dehydrogenase (GAPDH) quantification are as follows:

VP2 primer-probe set (Custom synthesis, IDT) probe: 5´-/5HEX/TGTTCTCCA/ZEN/CAATCTCCCAGGCTT/3IABkFQ/-3´ primer 1: 5´-CCTGGAGTGAATGCCTTTGT-3´ primer 2: 5´-AGAGGTTAAGGCTGGCAAATC-3´

GAPDH primer-probe set (cataloged as Hs.PT.39a.22214847; IDT) Probe: 5´-/56-FAM/TCATCCATG/ZEN/GTGAGCTGGCGG/3IABkFQ/-3´ primer 1: 5´-ACAGAGCCTCGCCTTTG-3´ primer 2: 5´-CCTTGCACATGCCGGAG-3´

For serotonin receptor expression analysis, total RNA was isolated from primary human pericytes, astrocytes, and choroid plexus epithelial cell culture lysates using an RNeasy Plus Mini Kit (Qiagen, #74134) following the manufacturer’s instructions. Real-time PCR was performed using the Bio-Rad iScript Reverse Transcription Supermix for RT-qPCR (Bio-Rad, Catalog #1708891) and TaqMan Fast Advanced Master Mix (ThermoFisher, #4444556). TaqMan gene expression assays were obtained from Thermo Fisher Scientific. RT-qPCR was performed with a Bio-Rad CFX96 detection system. TaqMan gene expression assays and 5-HT_2_A primer- Hs00167241_m1; 5-HT_2_B primer: Hs00168362_m1; 5-HT_2_C primers: Hs00968671_m1 and Hs00968672_m1. Expression levels of genes of interest were normalized to GAPDH as an internal control. Each reaction was performed in triplicate in three independent experiments.

### Statistical analysis

All experiments were performed in triplicate. Error bars represent the standard deviation, and means of triplicate experiments are shown. Significance (Fig. 2B and D) was determined using Student’s *t*-test two-tailed analysis and is denoted by * where *P* < 0.05. The relative expression of viral protein was calculated using the 2^–∆∆Ct^ method.

## RESULTS

### Cultured pericytes express characteristic markers *in vitro*

**Fig 1:**
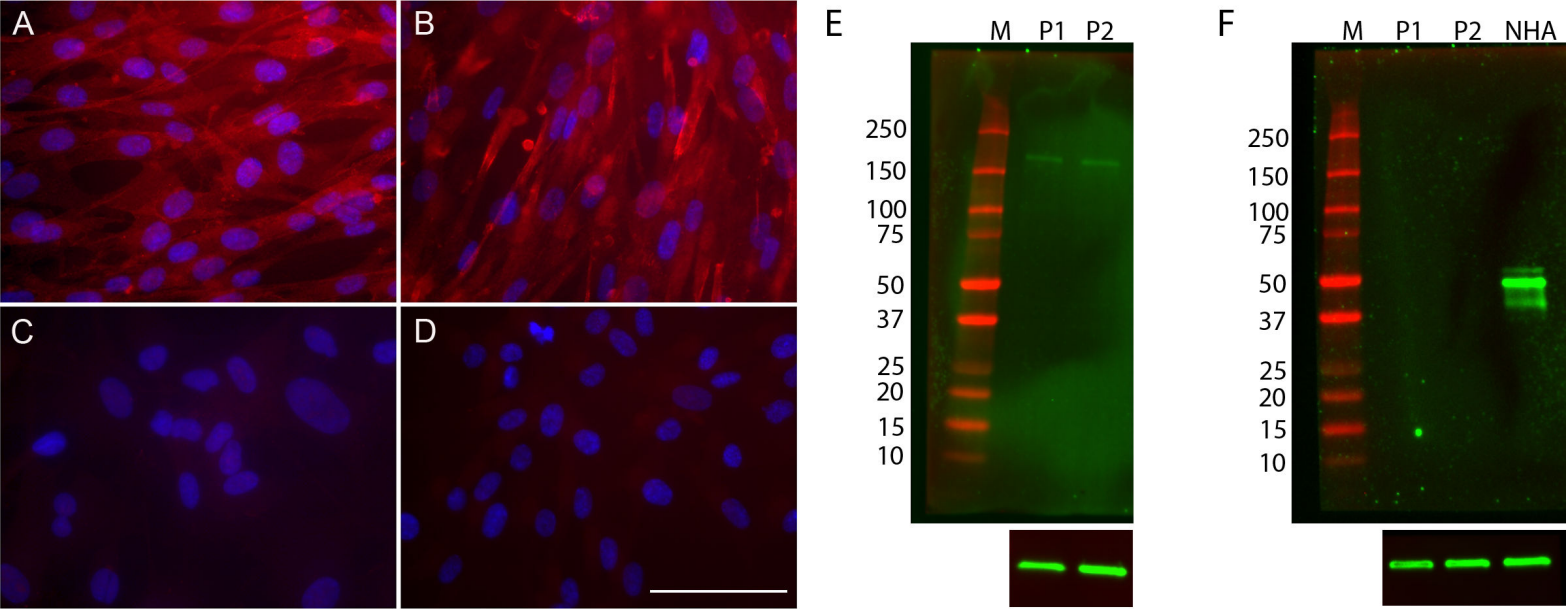
Primary human pericytes display characteristic markers. Cultured pericytes express the pericyte markers PDGFRβ (**A**) and NG2 (**B**). The cells are negative for the astrocyte marker GFAP (**C**), as well as cells stained with secondary antibody only (**D**). Scale bar = 100 µM. Representative western blot analyses show that pericytes derived from two different human pericyte donors express PDGFRβ (E; predicted molecular weight = 180 kDa) and do not express GFAP (**F**), while this antibody detects abundant GFAP at the predicted size of 50 kDa in normal human astrocytes. P1 = ScienCell Lot #34444; P2 = Lot #37047; NHA = normal human astrocytes. Actin was used as a loading control (lower panels).

To characterize the primary cultures of neurovascular pericytes, we stained the cells with known pericyte markers. Cells were positive for both PDGFRβ and neural/glial antigen-2 (NG2; Fig. 1A and B) and negative for the astrocyte marker GFAP as well as the secondary-only control (Fig. 1C and D), indicating that these cells are pericytes. While there are no markers exclusively specific for pericytes, the combination of PDGFRβ and NG2 expression is considered sufficient for pericyte identification (43). Primary pericytes were sourced from two different donors. To determine that the antibodies used in our immunofluorescence assays specifically recognize their designated antigens, we performed western blot analysis on protein lysates derived from two distinct pericyte donors, which confirmed the antibodies recognized their cognate antigens at the predicted molecular weight (Fig. 1E and F).

### Binding of JCPyV to pericytes is dependent on the presence of the viral attachment receptor LSTc

**Fig 2:**
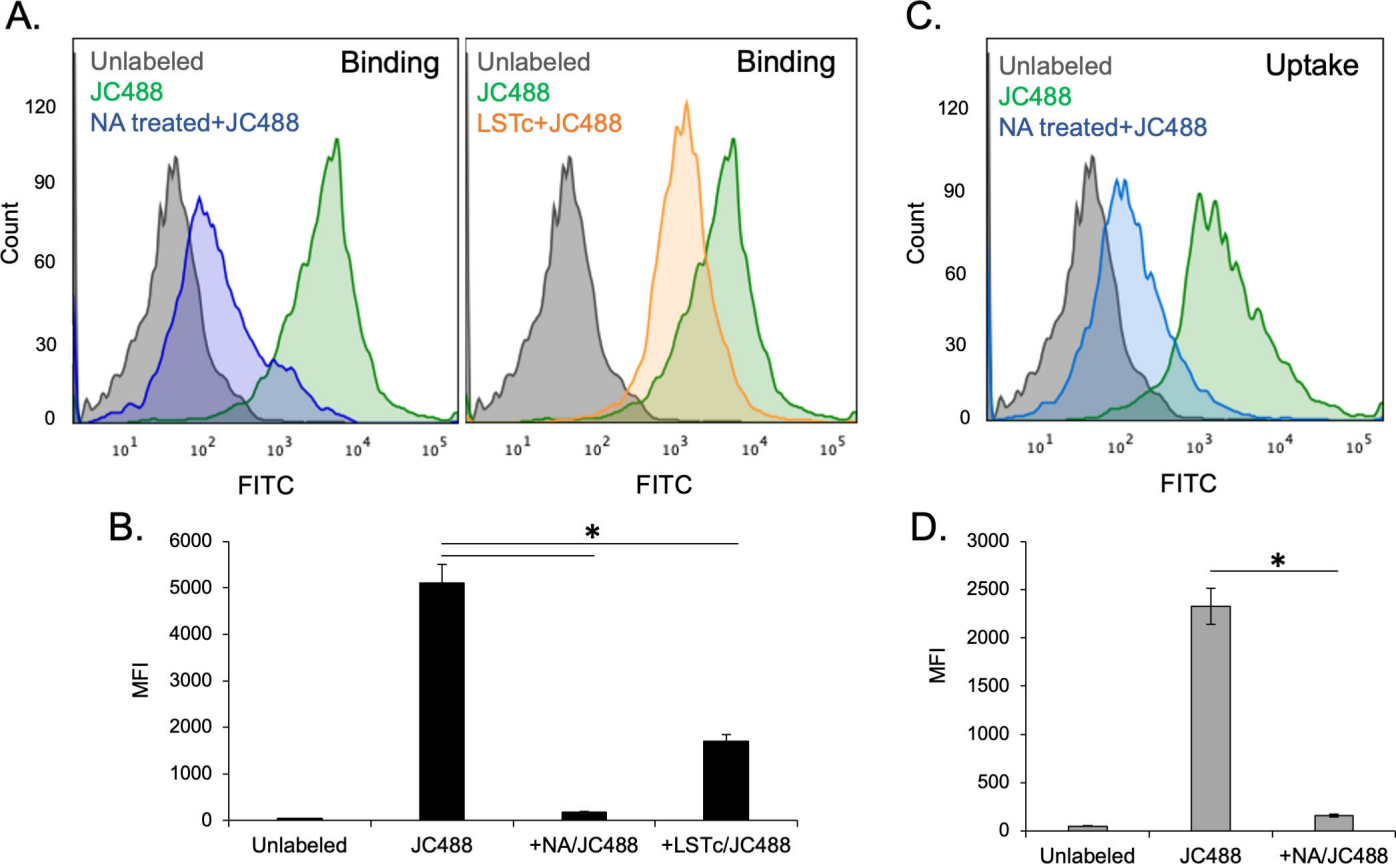
Flow cytometric characterization of JCPyV binding and internalization in primary pericytes. Pericytes were pretreated with type II neuraminidase (NA) for 1 h or pretreated with PBS control (no enzyme present). Cells treated with neuraminidase showed a significantly reduced ability to bind Mad1/SVEΔ−488 vs PBS-only treated cells (A, left panel). Mad1/SVEΔ−488 preincubation with 5 mM LSTc was also able to reduce viral binding to pericytes (A, right panel). The median fluorescence intensity (MFI) of NA vs PBS-treated cells bound to JMad1/SVEΔ−488, and of JCPyV-488/LSTc complexes bound to cells is shown. Both treatments are able to significantly reduce binding (**B**). Pericytes were pretreated with type II neuraminidase for 1 h or pretreated with PBS control (no enzyme present). Cells treated with neuraminidase showed a significantly reduced ability to internalize Mad1/SVEΔ−488 vs PBS-only treated cells (**C**). The MFI of NA vs PBS-treated cells in which Mad1/SVEΔ−488 was allowed to internalize for 1 h at 37°C following neuraminidase treatment (**D**). Error bars represent standard deviation. * *P* < 0.05

To determine the nature of initial virus engagement with the pericytes, cells were treated with neuraminidase to remove cell surface sialic acids. Neuraminidase treatment resulted in dramatically decreased AF488-labeled virus binding to the cell surface (Fig. 2A and B). In addition, AF488-labeled virus binding decreased in competition assays with LSTc, the specific VP1-engaging motif of the attachment receptor (Fig. 2A and B). In addition to inhibiting binding, cells pretreated with neuraminidase to remove surface sialic acid also showed a significantly reduced ability to internalize JCPyV compared to sialic acid-intact pericytes (Fig. 2C and D).

### JCPyV productively infects human pericytes

**Fig 3:**
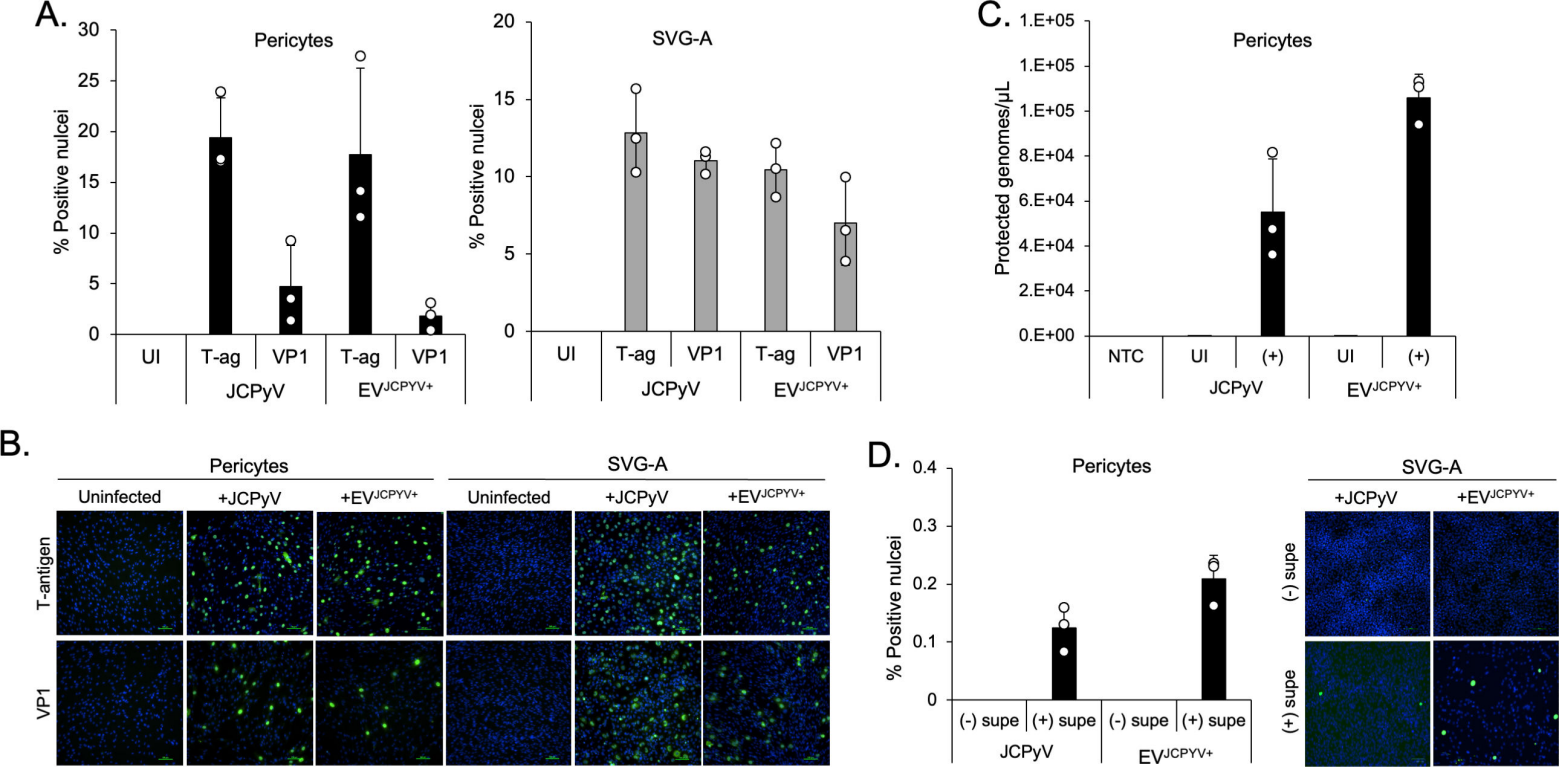
Primary human pericytes support JCPyV infection. Pericytes and control SVG-A cells were infected with purified Mad1/SVEΔ or infectious EV. (**A**) Infections in each cell type were scored at 3 days post-infection for the early and late viral proteins, T antigen and VP1. Error bars represent standard deviation; dots represent individual replicates. (**B**) Representative images of uninfected, Mad1/SVEΔ-infected, or EV-infected cells. DAPI is shown in blue and represents the total number of cells. T antigen^+^ and VP1^+^ nuclei are shown in green and represent infected cells. Scale bar = 100 nm. (**C**) Quantitative PCR of supernatants from virus or EV-infected pericytes. Pericytes were infected with Mad1/SVEΔ-purified JCPyV or virus-associated extracellular vesicles. Following infection with either form of JCPyV, pericytes release encapsidated virus into the supernatant at 3 days post-infection. Error bars represent the standard deviation; dots represent individual replicates. (**D**) Supernatants from infected (+) and uninfected (−) pericytes were used to reinfect SVG-A cells. Infectious EV and purified Mad1/SVEΔ were used as controls. Reinfections were fixed at 4 days post-infection and scored for VP1^+^ nuclei. Supernatants from infected pericytes contain infectious JCPyV.

To determine whether primary human pericytes are susceptible to infection with JCPyV *in vitro*, the cells from two different donors were exposed to either purified virus or infectious extracellular vesicles (JCPyV-EV) and assayed after 72 h by indirect immunofluorescence analysis. By 3 days post-infection, the virus established a productive infection in the pericytes, as shown by expression of both the early viral protein large T antigen and the late viral protein V antigen (VP1) as compared with the SVG-A glial cell line as an infection control (Fig. 3A and B). Pericytes infected with purified JCPyV or EV-associated JCPyV released encapsidated virus into the supernatant, as measured by qPCR (Fig. 3C). Virus harvested from the supernatants was capable of infecting naïve glial cells as quantified by the expression of T antigen and VP1 (Fig. 3D).

### Pericytes are permissive to different JCPyV strains

**Fig 4:**
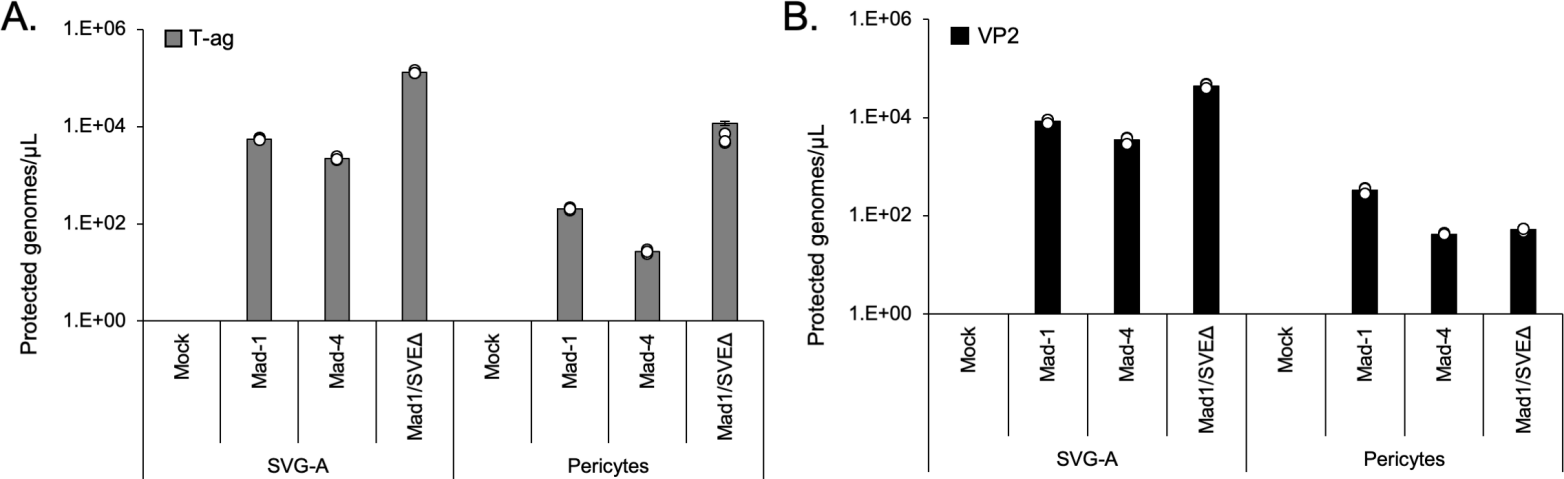
Primary pericytes express T antigen and VP2 following transfection from multiple JCPyV strains. Pericytes and control SVG-A cells were transfected with linearized Mad-1, Mad-4, Mad1/SVEΔ, or empty vector DNA. At 4 days post-transfection, culture supernatants were collected and analyzed by qPCR to quantify encapsidated viral genomes released into the media via T-ag (**A**) and VP2 (**B**) detection. All three strains of virus were detected in the supernatant at 4 days post-transfection. Control SVG-A cells released more encapsidated virus than pericytes.

The Mad1/SVE∆ strain of JCPyV, a Mad-1 genotype virus that contains a hybrid JCPyV/SV40 promoter, is typically used in our experiments. To investigate whether wild-type virus replication is supported in pericytes, we transfected cells with Mad-1 and Mad-4 plasmid as well as Mad1/SVE∆ plasmid. Because the only difference between these strains is in the non-coding control region, we chose to transfect infectious viral DNA rather than to infect with purified viruses. Viral release was quantified by qPCR using probes targeting T-ag (Fig. 4A) and VP2 (Fig. 4B). Pericytes release encapsidated genomes from all three strains into the supernatant.

### Pericytes express internalization receptors

**Fig 5:**
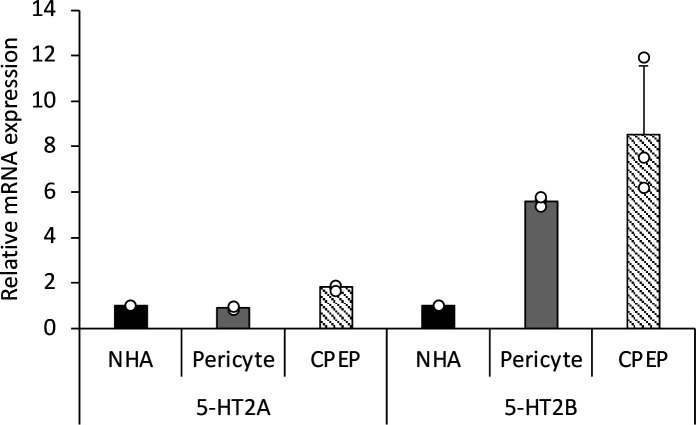
Expression of JCPyV internalization receptors in human brain pericytes. Pericytes express serotonin receptors 5-HT_2A_ and 5-HT_2B_ at levels comparable to control normal human astrocytes (NHA) and choroid plexus epithelial cells (CPEP). Receptor expression was determined by using RT-qPCR and normalized to endogenous GAPDH expression.

Because the pericytes were susceptible to JCPyV infection, we also tested them for expression of the 5-HT_2_ family of serotonin receptors, which have been identified as virus internalization receptors (11, 12, 14, 44). RT-qPCR analysis demonstrates that pericytes express 5-HT_2A_ and 5-HT_2B_ receptors at similar levels to primary human astrocytes and choroid plexus epithelial cells, two other known primary cell hosts for JCPyV (Fig. 5).

## DISCUSSION

**Fig 6:**
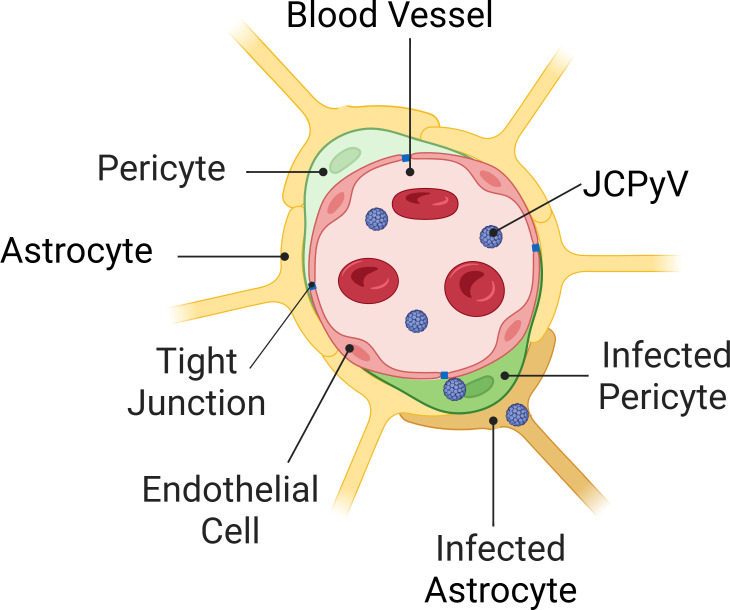
Model of the blood-brain barrier neurovascular unit. The blood-brain barrier is the interface between the blood and the brain that restricts entry of harmful substances to the CNS and is a potential site of neuroinvasion by JCPyV. Within the blood-brain barrier, blood vessels are surrounded by endothelium containing tight junctions, pericytes, and astrocyte endfeet. This cross-section of a blood vessel in the brain depicts the proximity of pericytes to astrocyte endfeet, thus highlighting the hypothesized path of infection of JCPyV through the brain endothelium, to the pericytes. Once a pericyte has been infected, we hypothesize the spread of infection to astrocytes, a target cell of JCPyV pathogenesis.

The blood-brain barrier is a multicellular structure that separates circulating blood from the central nervous system (Fig. 6). This physiological barrier is composed in part of endothelial cells that face the peripheral blood and are bound by tight junctions that restrict the entry of cells, molecules, and pathogens into the brain. The neuroendothelium shares a basement membrane on its abluminal side with pericytes, a specialized type of perivascular cell. The pericytes play a critical role in both the development and maintenance of the neuroendothelial barrier, by maintaining barrier stability and suppressing transcytosis (45). Transgenic mice deficient in pericytes show increased vascular permeability (38, 39, 46), demonstrating that pericytes are essential in the barrier functions of the BBB. Pericytes, in turn, are covered by astrocytic endfeet leading into the brain parenchyma (47).

In this work, we show that primary human pericytes from two different donors can be infected by the neurotropic polyomavirus JCPyV. Virus binding to pericytes is mediated by cell surface sialic acid, as attachment is abolished by pretreatment with neuraminidase. Binding of labeled virus is selectively competed by the presence of the sugar LSTc. Crystallographic evidence shows that LSTc is the known carbohydrate moiety that specifically interacts with the capsid protein VP1 of JCPyV (8, 9, 48). Our previous work has shown that, in addition to LSTc, JCPyV requires 5-HT_2_ serotonin receptors to enter the cell for productive infection (11, 12, 49). Cultured pericytes express 5-HT_2A_ and 5-HT_2B_ at similar levels compared with two other known host cell types, astrocytes and choroid plexus epithelial cells.

Despite the fact that pericytes are permissive to multiple strains of JCPyV, they are less permissive than the control glial cell line SVG-A. Pericytes and SVG-A cells expressed the early protein large T antigen at comparable levels, but pericytes produced diminished amounts of the late viral protein VP1. This could indicate that JCPyV infection is restricted in pericytes, which is similar to what we observed in kidney or choroid plexus epithelial cells (50–52). Interestingly, other neurotropic viruses that infect pericytes, such as severe acute respiratory syndrome coronavirus 2 (53–55) and HIV (56), also demonstrate measurable but relatively low levels of productive virus infection. Pericytes have been reported to be a target of infection by a variety of additional neuroinvasive viruses (reviewed in reference 57), including human cytomegalovirus (HCMV) (58), Zika (59, 60), and Powassan viruses (61). It has been hypothesized that barrier disruption following infection can allow additional virus into the brain, or, in the case of HIV, use the pericytes as a site of latency (42).

In order for JCPyV to cause PML, the virus must cross the barrier(s) that protect the brain to infect target glial cells. How JCPyV invades the CNS to cause disease is incompletely understood. We and others have shown that the virus as well as infectious extracellular vesicles can productively infect the cells that form the blood-cerebrospinal fluid barrier (BCSFB), the choroid plexus epithelium (52, 62–66). Viral entry across the BCSFB is therefore a plausible pathway into the CNS. In addition, we have demonstrated that JCPyV-infected choroid plexus cells secrete chemokines, including chemokine ligand 2 (CCL2) (67, 68), that are known to disrupt the endothelial integrity of the blood-brain barrier (67, 68), so it is possible that JCPyV accesses brain pericytes following an initial infection of the epithelial cells of the choroid plexus.

While the BCSFB appears to be an invasion route for JCPyV into the brain, relatively little attention has been paid to the potential of the BBB as a point of entry. Following primary infection, the virus is reactivated and spreads via viremia to the site of pathogenesis, the CNS. As early demyelinating lesions are frequently observed along the vasculature in brain sections of PML patients, it has been hypothesized that JCPyV can enter the brain through a hematogenous route by crossing the blood-brain barrier (21, 22, 35). We have recently observed that JCPyV does not infect brain endothelial cells. While there is an earlier report to this effect (69), we did not observe productive infection of brain endothelial cells using either the human cerebral microvascular endothelial cells (HCMEC) cell line or human induced pluripotent stem cell derived endothelial cells (iPSC-ECs) (36). We did, however, observe in our *in vitro* barrier system that brain endothelial cells can internalize virus, and a small amount of infectious virus can penetrate the endothelial monolayer, which suggests that virus could encounter cells on the abluminal side of the endothelium. The lack of productive infection of brain endothelial cells led us to examine pericytes, another essential cell type of the BBB, which until now had not been investigated as a host for JCPyV. Pericytes are closely apposed/in close contact with astrocytes, a main pathogenic target of JCPyV. Thus, if the pericytes are infected, it could easily spread to astrocytes. Here, we show for the first time that an additional barrier cell type involved in protecting the brain from invasion is susceptible to virus infection and suggests that JCPyV can enter the brain via the blood-brain barrier.
